# The Unfolding Counter-Transition in Rural South Africa: Mortality and Cause of Death, 1994–2009

**DOI:** 10.1371/journal.pone.0100420

**Published:** 2014-06-24

**Authors:** Brian Houle, Samuel J. Clark, F. Xavier Gómez-Olivé, Kathleen Kahn, Stephen M. Tollman

**Affiliations:** 1 Department of Sociology, University of Washington, Seattle, Washington, United States of America; 2 Institute of Behavioral Science (IBS), University of Colorado at Boulder, Boulder, Colorado, United States of America; 3 MRC/Wits Rural Public Health and Health Transitions Research Unit (Agincourt), School of Public Health, Faculty of Health Sciences, University of the Witwatersrand, Johannesburg, South Africa; 4 Centre for Global Health Research, Umeå University, Umeå, Sweden; 5 INDEPTH Network, Accra, Ghana; 6 ALPHA Network, London, United Kingdom; Institute of Infectious Diseases and Molecular Medicine, South Africa

## Abstract

The HIV pandemic has led to dramatic increases and inequalities in adult mortality, and the diffusion of antiretroviral treatment, together with demographic and socioeconomic shifts in sub-Saharan Africa, has further changed mortality patterns. We describe all-cause and cause-specific mortality patterns in rural South Africa, analyzing data from the Agincourt health and socio-demographic surveillance system from 1994 to 2009 for those aged 5 years and older. Mortality increased during that period, particularly after 2002 for ages 30–69. HIV/AIDS and TB deaths increased and recently plateaued at high levels in people under age 60. Noncommunicable disease deaths increased among those under 60, and recently also increased among those over 60. There was an inverse gradient between mortality and household SES, particularly for deaths due to HIV/AIDS and TB and noncommunicable diseases. A smaller and less consistent gradient emerged for deaths due to other communicable diseases. Deaths due to injuries remained an important mortality risk for males but did not vary by SES. Rural South Africa continues to have a high burden of HIV/AIDS and TB mortality while deaths from noncommunicable diseases have increased, and both of these cause-categories show social inequalities in mortality.

## Introduction

The HIV pandemic has dramatically increased adult mortality, particularly in sub-Saharan Africa. In 2007, South Africa accounted for 17% of the global burden of HIV infection [1], [2]. At the same time, demographic change and the diffusion of antiretroviral therapy [3] have decreased observed deaths in populations most at risk of HIV-related deaths [4], [5] and have increased life expectancy of people living with HIV [6]–[8]. These factors may produce an aging effect on the HIV epidemic with more older people suffering from HIV [9] and HIV+ adults reaching older ages where they face greater risk for noncommunicable diseases [10], [11]. Moreover, HIV may mask risk factors for cardiovascular disease as people infected with HIV generally have lower BMI and systolic blood pressure [12]. Over 80% of chronic disease occurs in low- and middle-income countries [13]; the burden in South Africa is two to three times that of industrialised countries given the burden of both communicable and noncommunicable diseases [10], [14]. This dual burden suggests a need for greater quantification in order to better describe trends and target interventions.

In Omran's original formulation of the epidemiological transition, countries moved from high mortality due largely to malnutrition and infectious disease to steadily declining mortality peaking at older ages and due largely to noncommunicable diseases [15]. Further refinements have called for two new stages: one in which age-specific death rates due to degenerative diseases are reduced and shift to older ages along with the increasing importance of lifestyle and personal behavior [16], [17]; and another to account for the emergence of new infectious diseases and re-emergence of old ones [18]. These transition trajectories also show variation in the pattern, pace and determinants of changes in cause-of-death patterns [15]. A classic transition shows progressive mortality and fertility declines followed by socioeconomic development. An accelerated transition starts later and is more rapid due to socioeconomic advances and enhanced by developments in medical and public health technologies. A delayed transition remains unfinished, incomplete with uneven socioeconomic development, and is largely driven by the spread of medical and public health interventions. Transition trajectories may also reflect polarization with marked inequalities in causes of death [19], [20]. Similarly, transitions may show patterns of convergence and divergence as changes occur, with divergence where certain social groups are most affected while others show dramatic gains that eventually converge [21].

However, epidemiological transition theory has several limitations. First, it suggests a linear, sequential pattern of stages when in fact these periods may overlap [22], patterns may reverse, or changes may remain incomplete [19] – with persisting co-existence of infectious and noncommunicable diseases. A continuing conceptual advance is the health transition [19], which seeks to incorporate the social and behavioral changes that may drive changes in mortality, fertility, and patterns of illness and death. However, it remains difficult to predict changing patterns of disease precipitated by development and may not adequately account for the major social and economic changes driving transitions. The Global Burden of Disease 2010 study highlighted both the extent of regional variation in transition patterns, and the lack of reliable data available to analyze these variations, particularly in rural areas [23]. What is needed is empirical information on the direction and scale of change at local, national, and regional levels.

Several studies of mortality patterns in South Africa have found evidence of a counter–epidemiological transition [24] due to the HIV pandemic increasing mortality in children and most adult age groups. Using data from the Agincourt demographic surveillance site, Kahn et al. [25] compared 1992–1993 to 2002–2003. They found dramatic increases in mortality for both sexes, particularly for ages 20–49 years. A further study refined these results by showing that recently returned migrants in the later period (2002–2003) were far more likely to die, and that the predominant cause-of-death was HIV/AIDS and tuberculosis [26]. Another study from the rural Agincourt sub-district, comparing 1992–1994 to 2002–2005, showed a modest increase in mortality from noncommunicable diseases in addition to the continued rise in mortality from chronic infectious disease [27]. Using data from a demographic surveillance system in KwaZulu-Natal Province, other researchers showed a high burden of deaths due to HIV in younger people and a concomitant burden of noncommunicable disease in older people [28].

While adult mortality has declined globally, the rise in parts of sub-Saharan Africa shows extreme differences among social groups. However, there is relatively little evidence on the upstream effects of household socioeconomic status (SES) on adult mortality in areas where HIV is endemic. Studies in industrialised countries have shown that relatively higher SES increases survival [29], [30]. Studies in sub-Saharan Africa have been limited by lack of adequate mortality data. Evidence suggests that SES is associated with health status and access to health services [31], but the effects of SES on adult mortality remain unclear. In Kwazulu-Natal [32] adult socioeconomic status was not significantly associated with adult mortality.

Using a longitudinal multivariate framework, we provide a detailed analysis of mortality patterns for those aged five years and older in a rural South African setting, analyses of child mortality (ages 0–4 years) are presented elsewhere [33], [34]. We aim to describe how all-cause mortality patterns vary by sex, age, time and household SES; and assess variation in cause-specific mortality patterns. This will help characterize the shifting burden of mortality over time in a region experiencing dramatic epidemiological and social change. Detailed longitudinal information may also help to effectively target limited resources and identify inequalities in mortality burden.

## Methods

### Ethics Statement

The Agincourt health and socio-demographic surveillance system (HDSS) was reviewed and approved by the Committee for Research on Human Subjects (Medical) of the University of the Witwatersrand (protocol M960720 and M081145). Community consent from civic and traditional leadership was secured at the start of surveillance and is reaffirmed from time to time, while informed verbal consent is obtained at individual and household level at each annual follow-up visit and approved by the ethics committees. A record of participant consent is kept of the household respondent who consented to interview as well as the responsible fieldworker. This information is captured on each household roster (populated census form).

### Data

We use data from the HDSS to describe the population living in the Agincourt subdistrict of Bushbuckridge, Mpumalanga Province, South Africa [35], [36]. The study area can be considered a border region of rural Southern Africa as well as South Africa: over 30% of the population are Mozambican immigrants, formerly refugees who entered the area following the Mozambican war and share family and kinship ties with the host South African population [35]. The data contain records of some 82,000 people (the exact number changes over time) living in 21 rural villages. Trained fieldworkers collect data by interviewing the most knowledgeable person in each household, including vital events (deaths, births), spatial movements, nuptial events and an array of other information. We use data from 1994 through 2009.

Household socioeconomic status has been collected every two years since 2001 using a 34-item asset status survey [37]. The survey covers the living conditions and assets of the household, including factors such as access to water and electricity, and ownership of appliances, transport, and livestock. Each variable is normalized to have range zero to one. An index is created by categorizing assets into five groups: modern assets, livestock assets, power supply, water and sanitation, and dwelling structure. The scaled asset values are summed within each group and then scaled again on [0,1]. Finally, these five asset groups are summed to yield an overall asset score for each household in the range of zero to five. We operationalize household socioeconomic status by taking quintiles of this asset score over all the years in which it was collected.

Our cause-specific models use cause-of-death information from verbal autopsy (VA) interviews. For every death recorded in an annual census round, trained lay fieldworkers interview the closest relative of the deceased person, using a standardized, validated instrument and approach [36], [38]. To identify the most likely cause of death, we analyze this information using InterVA-4 (http://www.interva.net), a Bayesian probability model for interpreting VA data [39], [40]. We specify the model to suit a local setting with high prevalence of HIV and very low prevalence of malaria. We have used InterVA-4 rather than a physician-based VA approach because InterVA-4 ensures consistent coding and hence comparability across time, whereas physician coders change periodically. A complementary analysis using physician-based assessments produced similar findings. Indeterminate causes of death were not included in the cause-specific analysis – these individuals were considered censored at the time of their death. An alternate model including these indeterminate causes as a separate cause of death category yielded similar findings.

### Statistical Analysis

We model mortality using discrete time event history analysis [41] for non-repeating events [42]. Data are organized as person-years with one record for each fully-observed year lived by each person aged 5+ years, including the year they died if there was a death. Values for covariates are set *at the beginning* of each person year, and the death indicator is set to ‘1’ if there was a death during the year. We use logistic regression to estimate the yearly probability of dying from all possible causes and the relative importance of sex, age, time period, and household SES as predictors of death during a year. We use multinomial logistic regression to estimate the yearly probability of dying by specific cause-of-death categories and include covariates sex, age, time period, and household SES. Interactions between predictor variables are tested using likelihood ratio tests for nested models. Time periods are selected to simultaneously maximize our ability to detect change along the various dimensions included in the models, while acknowledging the fundamental dynamics of the HIV epidemic in the study population. HIV began affecting mortality in the period 1997–8, and consequently we include a break-point in the time periods between 1997 and 1998. For most models we use relatively wide four-year intervals to capture temporal change because this ensures sufficient deaths in each cell. For the SES model we use two-year time intervals because SES is updated on a two-year cycle. We categorize causes of death into four groups based on the Global Burden of Disease Study [43]: (1) HIV/AIDS and TB; (2) other communicable, maternal, perinatal, and nutritional diseases (excluding HIV/AIDS and TB); (3) noncommunicable diseases; and (4) injuries. Group 1 includes HIV *and* TB because HIV is an underlying cause in most TB deaths and the VA method does not easily distinguish HIV-related from non-HIV-related TB deaths. Group 2 includes among others diarrhea, malaria, and respiratory diseases. Group 3 includes cancer, cardiovascular disease, congenital diseases, diabetes, epilepsy, kidney disease, liver disease, and upper gastrointestinal bleeds. Group 4 involves injuries from accidents (including transport accidents), assault, suicide, and other external causes. SES models are restricted to years 2001–2009. We estimate all models using Stata [44]. We summarize these models using predictive probabilities for discrete sex-age-period groups. Differences over time for each sex-age group are compared relative to the referent (earliest) time period.

### Data Availability

Full documentation associated with the Agincourt HDSS, as well as an anonymized 10% sample of the full database are available at the Agincourt data website (http://www.agincourt.co.za/). Customized data extraction can be requested from Dr. F. Xavier Gómez-Olivé (Xavier@agincourt.co.za). Full details of data sharing and collaborations are detailed elsewhere [35].

## Results

### All-Cause Mortality

Demographic characteristics for the all-cause estimation sample are shown in Table 1 under the column “All-Cause” (person-year counts are shown in Table S1). Estimation results from the all-cause model are presented in Table S2. We estimated a full model including sex, age, and time. Interactions between sex and age (p<0.001), age and time (p<0.001), and sex and time (p = 0.009) significantly improved model fit. A model with these two-way interactions was tested against the same model with a three-way interaction among sex, age, and time; the latter model again significantly improved model fit (p<0.001) and constituted the final model (Table S2).

**Table 1:** Demographic characteristics of “all-cause” and “cause-specific” estimation samples, Agincourt, South Africa, 1994–2009.

	All-Cause		Cause-Specific	
Variable	Overall	SES	Overall	SES
	(n = 123,858)	(n = 40,573)	(n = 123,448)	(n = 40,395)
*Sex*				
Male	56,018	16,953	55,802	16,860
Female	67,840	23,620	67,646	23,535
Mean Age: years (Std. Dev.)	27.16 (18.21)	27.92 (18.33)	27.12 (18.18)	27.88 (18.30)
Mean Age at Death: years (Std. Dev.)	49.34 (21.17)	48.16 (20.62)	48.83 (20.88)	47.83 (20.38)
Number of Deaths Ages 5+	8,262	5,636	6,896	4,791
*Deaths by Cause*				
HIV/AIDS and TB	–	–	3,742	2,779
Other Communicable Causes	–	–	592	358
Noncommunicable Causes	–	–	1,971	1,297
Injuries	–	–	591	357
*Deaths by Period*				
1994–1997	1,123	–	880	–
1998–2001	1,635	352^a^	1,324	284^a^
2002–2005	2,713	2,605	2,318	2,225
2006–2009	2,791	2,679	2,374	2,282

a SES measurements began in 2001, restricting the estimation sample to 2001–2009.

**Figure 1:**
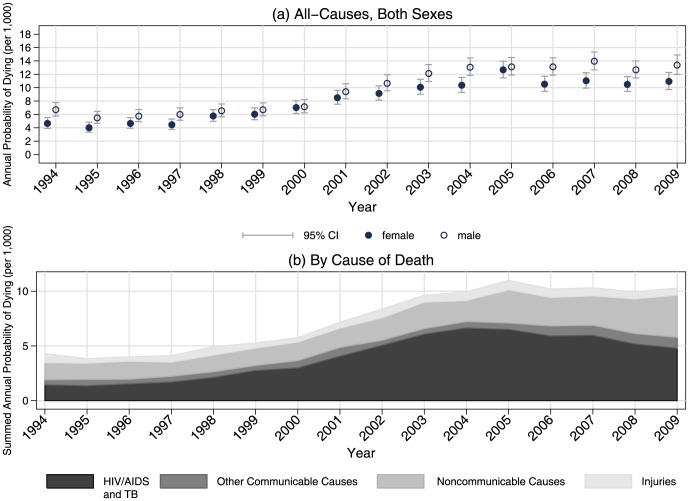
Annual probability of dying by (a) all-causes and (b) specific causes, Agincourt, South Africa, 1994–2009.

We first describe the all-cause mortality patterns by time, using bivariate logistic regression. Figure 1, panel (a) shows the predicted annual probability of dying per 1,000 by year, stratified by sex. The probability of dying has increased over time, especially since 2002. Since 2005 mortality has begun to plateau.

**Figure 2:**
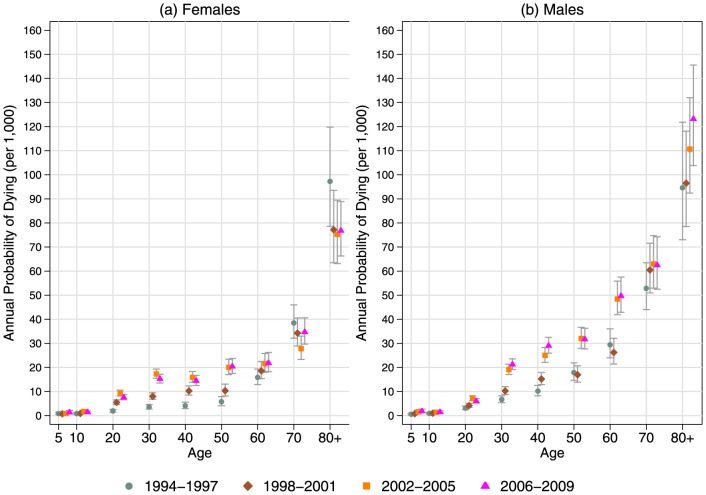
Annual probability of dying by age and time, (a) females and (b) males, Agincourt, South Africa, 1994–2009.

The odds ratios in Table S2 describe the changes in a person's probability of dying as a function of sex, age, and time period. The actual annual probabilities of dying associated with these odds ratios are presented in Figure 2. For both males and females the probability of dying has increased over time. For females (panel [a]), the probability of dying began to increase (relative to 1994–1997) starting in 1998–2001 for ages 20–49 (p<0.001) and 50–59 (p = 0.004). However, the greatest increases were after 2002 for ages 30–59 (p<0.001). Again compared to 1994–1997, the probability of dying for those aged 60–69 increased in 2002–2005 (p = 0.005) and 2006–2009 (p = 0.004).

For males (panel [b]), the probability of dying began to increase starting in 1998–2001 for ages 20–29 (p = 0.016) and 30–49 (p<0.001). As with females, the largest increases came after 2002 for ages 30–69 but were more dramatic among males.

**Figure 3:**
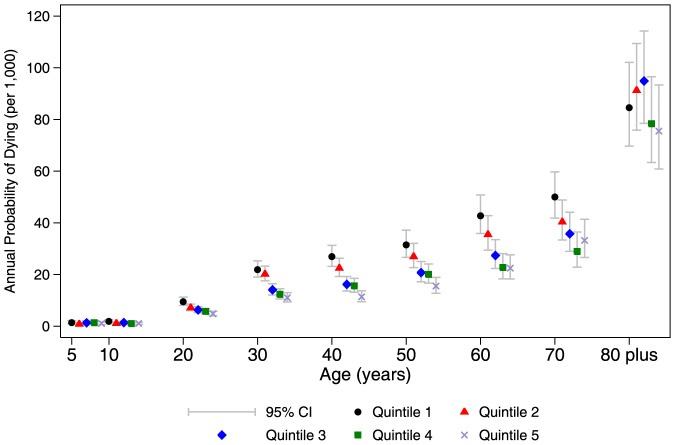
Annual probability of dying by household socioeconomic status, by age, Agincourt, South Africa, 2001–2009.

We next fit a model including household SES, sex, age, and time, allowing for a significant interaction between SES and age (p = 0.018; Table S3). Figure 3 plots the predicted probability of dying by quintiles of household SES over age. Those in the highest (fifth) SES quintile were at the lowest risk of dying until ages 70 and over. Conversely, those in the lowest (first) SES quintile were at the highest risk of dying until ages 80 and over. There was some variation by age for the second and fourth SES quintiles, but overall a clear inverse gradient between SES and mortality emerged for most ages except the very young and very old.

### Cause-Specific Mortality

Demographic characteristics for the cause-of-death estimation sample are shown in Table 1 under the column “Cause-Specific” (person-year counts are shown in Table S1). Estimation results from the cause-specific model are presented in Table S4. We estimated a full model including sex, age, and time. Interactions between sex and age (p<0.001), age and time (p<0.001), and sex and time (p = 0.023) significantly improved model fit and were included in the final model (Table S4). The referent group is surviving persons.

We first describe the cause-specific mortality patterns by time using bivariate multinomial logistic regression. Panel (b) in Figure 1 shows the predicted, summed annual probability of dying per 1,000 by year and cause-of-death. The probability for each cause is added to that of the other causes, and thus the cumulative area for successive curves represents the probability of dying from these causes in a given year. The probability of dying from HIV/AIDS and TB increased dramatically after 2000 but has plateaued since a peak in 2004–2005. Noncommunicable diseases, the next largest cause-of-death, have increased steadily over time.

**Figure 4:**
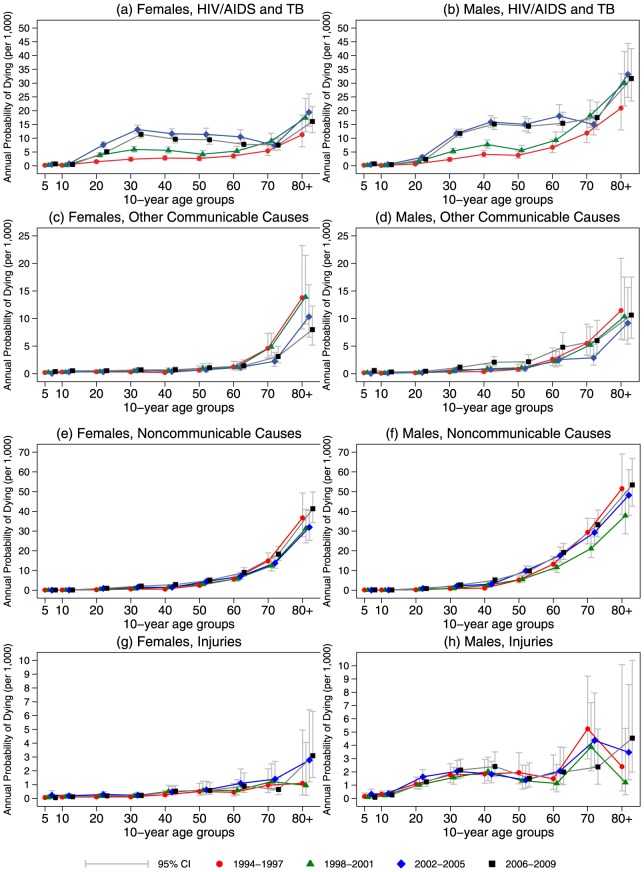
Annual probability of dying by cause of death, Agincourt, South Africa, 1994–2009.

The odds ratios in Table S4 describe the changes in a person's probability of dying from different causes as a function of sex, age, and time period. Figure 4 shows the actual annual probabilities of dying associated with these odds ratios, by cause and age, over time.

For HIV/AIDS and TB (panels [a] and [b] in Figure 4), there have been large increases in the probability of dying for both sexes since 2002, but mortality risk plateaued at high levels in 2006–2009. Across all periods, for females (panel [a]), the risk of dying peaked at ages 30–39 and then diminished. For males (panel [b]), the risk of dying remained high throughout ages 30–69.

For other communicable diseases, including maternal, perinatal, and nutritional causes but excluding HIV/AIDS and TB (panels [c] and [d] in Figure 4), the probability of dying was similar and low for both females (panel [c]) and males (panel [d]).

For noncommunicable diseases (panels [e] and [f] in Figure 4), the probability of dying for both females (panel [e]) and males (panel [f]) has slowly increased over time periods relative to 1994–1997, particularly since 2002. For both females and males aged 20–59, compared with 1994–1997, the probability of dying increased significantly in 2002–2005 and 2006–2009. In 2006–2009 there were also significant increases for ages 60–69 for both females and males (again, compared with 1994–1997). In general, the probability of dying from noncommunicable diseases was higher among males than females.

For injuries (panels [g]) and [h] in Figure 4), the probability of dying for females (panel [g]) remained low over each time period. Males (panel [h]) were at greater risk of dying from injuries, particularly after age 20. There was little systematic variation over time for either sex.

We next fit a model including household SES, sex, age, and time. Interactions between SES and the other covariates did not significantly affect model fit (Table S5).

**Figure 5:**
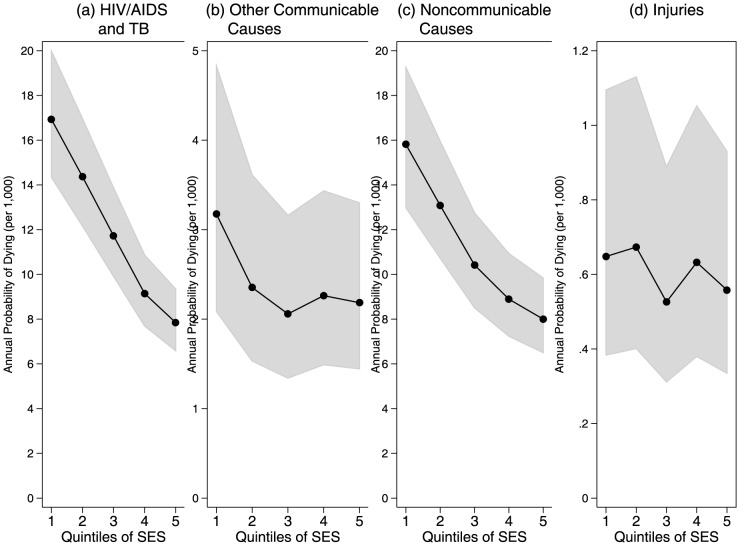
Annual probability of dying by cause of death and household socioeconomic status, Agincourt, South Africa, 2001–2009.

Figure 5 plots the predicted probability of dying by quintiles of household SES for each cause-of-death group. There was a general inverse gradient between household SES and mortality for most cause types. However, the consistency of the pattern varied considerably between causes. There were strong inverse gradients for those dying from HIV/AIDS and TB (panel [a]) and from noncommunicable diseases (panel [c]). A smaller and less consistent inverse gradient emerged for those dying of communicable diseases other than HIV/AIDS and TB (panel [b]). There was no pattern relating SES to deaths from injuries (panel [d]).

## Discussion

Several factors may explain these mortality trends. Antiretroviral therapy (ART) only became available in the three hospitals surrounding the study area in 2004/2005 [45]. Unpublished work describing data collected from the voluntary counseling and testing (VCT) and HIV services of primary health care facilities in the study site indicates that between August 2006 and March 2009 a total of 7,738 clients were recorded (Chodziwadziwa, personal communication). These facilities are all within 10 km of each Agincourt household. Beginning in 2007, antiretroviral therapy (ART) became available from clinics inside the study area, and by February 2009, 1,500 patients were on ART provided by public sector clinics [46]. There is no charge for VCT or ART. The plateau in HIV/AIDS and TB mortality during 2006–2009 likely reflects the impact of this ART rollout [47]. Although AIDS mortality may have decreased, the survival of increasing numbers of older people may help explain the rising mortality from noncommunicable diseases (NCDs) which share behavioral and lifestyle risk factors that are prevalent in South Africa [10].

Clearly, epidemiological polarization is evident in rural north-eastern South Africa, where the poor are at increased mortality risk due to two prominent and contrasting categories of causes-of-death. The poorest households are at higher risk of death (excepting in the very young and very old) other than for deaths due to injuries. Previous research has shown a positive relationship between HIV seroprevalence and socio-economic status (SES) [48]; our finding of an inverse gradient between SES and HIV/AIDS and TB deaths suggests that poorer households are at increased risk of the upstream effects of SES on mortality. Further, a cross-sectional study of HIV prevalence in Agincourt found a lower probability of being HIV+ among those living in households in the wealthiest SES quintile relative to the poorest quintile [49]. While ART became available in 2004 at district hospitals, many barriers to access persisted, including long waiting times, limited drug supplies, long travel distances and unaffordable transportation [50].

Poorer households lack the resources to care for HIV-infected adults; in turn, the loss of a healthy adult contributes to household poverty [51]. Research in Agincourt supports these findings: impoverished households are more likely to suffer an AIDS-related death and this is associated with subsequent declines in household SES [52]. Research also suggests that while income support (pensions for example) is available, the poorest households are less likely to receive these grants [53]. Similarly, the inverse gradient between SES and NCD deaths may reflect barriers to care. Transport and opportunity costs to manage a chronic condition are greater for the rural poor, and technologies are often concentrated in hospitals that are more difficult to reach. A pattern emerges in which the rural poor are burdened by NCDs, lack access to care, and are further impoverished by the cost of care; increased mortality results [54]–[56].

Temporary migration is prevalent, especially among young adults and increasingly women and has complex associations with mortality [57]. Recently returned migrants were found to have a higher risk of dying compared with permanent residents or less recent migrant returnees, likely due to advancing illness particularly due to HIV/AIDS and TB [26], [57]. Long-distance migrants that return once or twice a year report many sexual partners, leading to increased risk of HIV infection for their partners [58].

In the context of profound demographic and epidemiological change, this study provides critical information on the nature of transitions underway in a rural border region of South Africa undergoing dramatic social change [25]. The Agincourt area is experiencing a counter-transition, with markedly increased all-cause mortality which appears to have recently plateaued. The transition is prolonged, reflected in a dual burden of disease and the presence of contrasting stages of transition. The high prevalence of HIV among older persons (16% of women and 18% of men 50 years and older were HIV+ [49]) suggests increasing co-morbidity between HIV/TB and NCDs and a likely increase in the demand for chronic care. [23] The pooling of mortality data from the many health and demographic surveillance sites making up the INDEPTH Network (International Network for the Demographic Evaluation of Populations and Their Health; www.indepth-network.org) [59] will permit a broad-ranging assessment of epidemiological transitions across a diversity of low- and middle-income settings and could contribute to refinements in transition theory.

These findings complement and extend the results of a recent study of the Agincourt HDSS examining the spatial distribution of adult mortality under a similar time span [60]. While using a different analytical approach they showed a similar temporal trend in mortality. Our study expands upon these findings by examining mortality trends across detailed sex-age groups including younger and older age groups. They also found an inverse gradient between household SES for all-cause mortality [60], while this study confirms that finding using a different operationalization of the SES measure, as well as summarizing variation in SES mortality across age groups and showing similar gradients for deaths due to HIV/AIDS and TB and noncommunicable diseases.

We acknowledge several limitations. Although we used an unusually robust and extensive dataset, data are from a defined geographic region within South Africa; further studies are needed to generalize to other settings although comparable mortality data shows similar patterns elsewhere in South Africa and sub-Saharan Africa [61]. A recent analysis in rural KwaZulu-Natal, a setting with very high HIV prevalence, found an increase in adult life expectancy of 11.3-years in 2011 compared to 2003, the year before public-sector ART became available [6]. Second, we used an asset-based approach to assess household SES; other approaches may clarify how different dimensions of SES affect mortality risk. Third, carrying the previous SES measure forward likely underestimates our standard errors. Fourth, as the data do not include an individual measure of HIV seroprevalence, it is difficult to determine whether excess deaths from AIDS among the poor result specifically from their increased risk of infection. Furthermore, while we focused on population-averaged mortality patterns over sex, age and time period, additional individual, household and community-level determinants may be important to include in future analyses (e.g., village-level space-time risk [60]). This focus on the population-averaged probability of dying also ignores potential unobserved heterogeneity at the individual level – particularly in terms of survivorship bias. Care must be taken to interpret these results at the population-level (i.e. for the group defined by each cell in the model, rather than each individual in the group). Unobserved heterogeneity at the individual level may reduce the magnitude of our estimated coefficients and predicted probabilities for temporal effects (i.e. make them conservative), but valid statistical hypothesis testing is still possible, see [42]. Consequential unobserved heterogeneity produces effects that decrease with time, and since most of the temporal effects we estimate *increase* with time, we are confident that the effects definitely do increase with time, and if unobserved heterogeneity is affecting them, they are actually larger than we estimate.

Rural South Africa continues to experience a counter-transition in mortality due to HIV/AIDS and TB. Concurrently, a dual burden of mortality is evident, with NCDs contributing to deaths among working-age and older persons. These mortality categories are disproportionately represented among poor households. Programmes are required to coordinate health care among chronic conditions, [62] whether infection or noncommunicable [10], [27], [63]. These would help limit duplication of chronic care services (reducing overall cost) and reveal synergies in care for patients with multiple chronic conditions, thus contributing to improved overall outcomes and, reduced risk among vulnerable households [64].

## Supporting Information

Table S1 **Person-Years by Age, Sex, Time Period, and Outcome, Agincourt, South Africa, 1994–2009.**
(DOCX)

Table S2 **Logistic regression of all-cause mortality, Agincourt, South Africa, 1994–2009.**
(DOCX)

Table S3 **Logistic regression of all-cause mortality by household SES, Agincourt, South Africa, 2001–2009.**
(DOCX)

Table S4 **Multinomial logistic regression of cause-specific mortality, Agincourt, South Africa, 1994–2009.**
(DOCX)

Table S5 **Multinomial logistic regression of cause-specific mortality by household SES, Agincourt, South Africa, 2001–2009.**
(DOCX)
