# Supplementary material for: The Unfolding Counter-Transition in Rural South Africa: Mortality and Cause of Death, 1994–2009
Source: PLoS One. 2014 Jun 24;9(6):e100420. doi: 10.1371/journal.pone.0100420 (PMC4068997; doi:10.1371/journal.pone.0100420)
Supplement: Table S1 — Person-Years by Age, Sex, Time Period, and Outcome, Agincourt, South Africa, 1994–2009. (DOCX) [file pone.0100420.s001.docx]

|  | 5–9 | 10–19 | 20–29 | 30–39 | 40–49 | 50–59 | 60–69 | 70–79 | 80+ |
| --- | --- | --- | --- | --- | --- | --- | --- | --- | --- |
| **Females** |  |  |  |  |  |  |  |  |  |
| **1994–1997** |  |  |  |  |  |  |  |  |  |
| Person years | 19185 | 30471 | 21823 | 15242 | 9567 | 6287 | 5822 | 2990 | 802 |
| All deaths | 17 | 26 | 42 | 54 | 39 | 36 | 92 | 115 | 78 |
| HIV/TB | 5 | 7 | 30 | 36 | 26 | 10 | 24 | 20 | 9 |
| Other communicable | 4 | 7 | 4 | 4 | 1 | 4 | 10 | 15 | 12 |
| Non-communicable | 3 | 4 | 3 | 6 | 3 | 10 | 32 | 49 | 33 |
| Injuries | 3 | 2 | 2 | 2 | 3 | 1 | 2 | 3 | 2 |
| **1998–2001** |  |  |  |  |  |  |  |  |  |
| Person years | 17752 | 32696 | 22643 | 16592 | 10821 | 6578 | 5543 | 3765 | 1218 |
| All deaths | 13 | 30 | 124 | 133 | 111 | 68 | 103 | 129 | 94 |
| HIV/TB | 5 | 8 | 91 | 97 | 60 | 28 | 31 | 29 | 18 |
| Other communicable | 5 | 7 | 10 | 5 | 10 | 5 | 5 | 20 | 15 |
| Non-communicable | 0 | 1 | 11 | 17 | 16 | 19 | 40 | 39 | 38 |
| Injuries | 1 | 5 | 3 | 1 | 4 | 4 | 3 | 8 | 1 |
| **2002–2005** |  |  |  |  |  |  |  |  |  |
| Person years | 16228 | 33731 | 24574 | 17332 | 12011 | 7460 | 5383 | 4467 | 1555 |
| All deaths | 14 | 58 | 228 | 300 | 191 | 149 | 116 | 124 | 117 |
| HIV/TB | 5 | 25 | 185 | 235 | 149 | 85 | 46 | 27 | 30 |
| Other communicable | 0 | 12 | 11 | 13 | 1 | 6 | 6 | 6 | 16 |
| Non-communicable | 0 | 4 | 19 | 25 | 16 | 31 | 37 | 69 | 46 |
| Injuries | 3 | 7 | 10 | 4 | 6 | 5 | 7 | 2 | 3 |
| **2006–2009** |  |  |  |  |  |  |  |  |  |
| Person years | 14526 | 31066 | 25897 | 17289 | 12311 | 7986 | 5208 | 4347 | 2148 |
| All deaths | 20 | 45 | 196 | 264 | 178 | 163 | 114 | 151 | 165 |
| HIV/TB | 11 | 12 | 132 | 191 | 110 | 80 | 45 | 37 | 32 |
| Other communicable | 5 | 19 | 13 | 13 | 9 | 9 | 6 | 13 | 14 |
| Non-communicable | 1 | 5 | 32 | 36 | 39 | 48 | 40 | 71 | 80 |
| Injuries | 0 | 3 | 3 | 5 | 7 | 6 | 4 | 3 | 6 |
| **Males** |  |  |  |  |  |  |  |  |  |
| **1994–1997** |  |  |  |  |  |  |  |  |  |
| Person years | 19479 | 31594 | 20856 | 13252 | 8465 | 5139 | 3090 | 2060 | 560 |
| All deaths | 11 | 29 | 65 | 88 | 86 | 92 | 91 | 109 | 53 |
| HIV/TB | 3 | 3 | 16 | 31 | 36 | 26 | 17 | 19 | 10 |
| Other communicable | 3 | 5 | 7 | 5 | 4 | 4 | 5 | 9 | 4 |
| Non-communicable | 1 | 2 | 6 | 14 | 11 | 32 | 41 | 53 | 22 |
| Injuries | 1 | 11 | 23 | 23 | 15 | 12 | 5 | 10 | 0 |
| **1998–2001** |  |  |  |  |  |  |  |  |  |
| Person years | 17830 | 33986 | 23499 | 14802 | 9210 | 5487 | 3469 | 2067 | 860 |
| All deaths | 14 | 34 | 98 | 152 | 140 | 93 | 91 | 125 | 83 |
| HIV/TB | 3 | 4 | 34 | 80 | 71 | 30 | 30 | 40 | 26 |
| Other communicable | 3 | 5 | 3 | 9 | 4 | 7 | 9 | 8 | 9 |
| Non-communicable | 2 | 6 | 10 | 10 | 24 | 32 | 32 | 49 | 29 |
| Injuries | 2 | 7 | 25 | 25 | 20 | 7 | 4 | 4 | 1 |
| **2002–2005** |  |  |  |  |  |  |  |  |  |
| Person years | 15975 | 34970 | 26044 | 15924 | 9829 | 6282 | 3656 | 1922 | 967 |
| All deaths | 25 | 49 | 186 | 304 | 246 | 201 | 177 | 121 | 107 |
| HIV/TB | 10 | 17 | 83 | 178 | 145 | 92 | 74 | 33 | 28 |
| Other communicable | 1 | 3 | 2 | 9 | 11 | 5 | 9 | 9 | 7 |
| Non-communicable | 1 | 3 | 28 | 35 | 31 | 62 | 63 | 46 | 45 |
| Injuries | 6 | 12 | 39 | 32 | 17 | 8 | 6 | 12 | 4 |
| **2006–2009** |  |  |  |  |  |  |  |  |  |
| Person years | 14032 | 31855 | 27599 | 16427 | 10088 | 6430 | 3421 | 1920 | 958 |
| All deaths | 26 | 48 | 167 | 349 | 293 | 204 | 170 | 120 | 118 |
| HIV/TB | 9 | 16 | 65 | 197 | 159 | 85 | 46 | 27 | 28 |
| Other communicable | 8 | 8 | 13 | 17 | 20 | 13 | 17 | 11 | 11 |
| Non-communicable | 3 | 3 | 17 | 44 | 46 | 53 | 70 | 69 | 53 |
| Injuries | 2 | 9 | 36 | 33 | 23 | 8 | 7 | 4 | 4 |
