# Supplementary material for: The Unfolding Counter-Transition in Rural South Africa: Mortality and Cause of Death, 1994–2009
Source: PLoS One. 2014 Jun 24;9(6):e100420. doi: 10.1371/journal.pone.0100420 (PMC4068997; doi:10.1371/journal.pone.0100420)
Supplement: Table S2 — Logistic regression of all-cause mortality, Agincourt, South Africa, 1994–2009. (DOCX) [file pone.0100420.s002.docx]

| Variable | Odds Ratio | 95% CI | p-value |
| --- | --- | --- | --- |
| *Sex* |  |  |  |
| Male | 0.763 | [0.405, 1.438] | 0.404 |
| *10-Year Age Groups* |  |  |  |
| 5–9 | 1.000 | – | – |
| 10–19 | 1.037 | [0.610, 1.762] | 0.894 |
| 20–29 | 1.985 | [1.202, 3.278] | 0.007 |
| 30–39 | 4.147 | [2.572, 6.687] | < 0.001 |
| 40–49 | 4.409 | [2.661, 7.307] | < 0.001 |
| 50–59 | 7.128 | [4.327, 11.742] | < 0.001 |
| 60–69 | 17.681 | [11.208, 27.892] | < 0.001 |
| 70–79 | 43.506 | [27.719, 68.286] | < 0.001 |
| 80+ | 116.151 | [72.832, 185.236] | < 0.001 |
| *Time Period* |  |  |  |
| 1994–1997 | – | – | – |
| 1998–2001 | 0.859 | [0.433, 1.706] | 0.664 |
| 2002–2005 | 1.012 | [0.518, 1.979] | 0.972 |
| 2006–2009 | 1.616 | [0.882, 2.962] | 0.12 |
| *Interactions between Sex and Age* |  |  |  |
| Male ***X*** age 10–19 | 1.479 | [0.682, 3.208] | 0.322 |
| Male ***X*** age 20–29 | 2.243 | [1.087, 4.631] | 0.029 |
| Male ***X*** age 30–39 | 2.189 | [1.086, 4.414] | 0.029 |
| Male ***X*** age 40–49 | 3.508 | [1.711, 7.190] | 0.001 |
| Male ***X*** age 50–59 | 3.624 | [1.775, 7.400] | < 0.001 |
| Male ***X*** age 60–69 | 2.462 | [1.242, 4.878] | 0.01 |
| Male ***X*** age 70–79 | 1.875 | [0.953, 3.690] | 0.069 |
| Male ***X*** age 80+ | 1.357 | [0.665, 2.767] | 0.402 |
| *Interactions between Sex and Time* |  |  |  |
| Male ***X*** 1998–2001 | 1.404 | [0.524, 3.763] | 0.499 |
| Male ***X*** 2002–2005 | 2.378 | [0.956, 5.911] | 0.062 |
| Male ***X*** 2005–2009 | 1.763 | [0.746, 4.171] | 0.197 |
| *Interactions between Age and Time* |  |  |  |
| Age 10–19 ***X*** 1998–2001 | 1.209 | [0.522, 2.800] | 0.658 |
| Age 10–19 ***X*** 2002–2005 | 1.924 | [0.874, 4.236] | 0.104 |
| Age 10–19 ***X*** 2005–2009 | 1.015 | [0.480, 2.144] | 0.969 |
| Age 20–29 ***X*** 1998–2001 | 3.785 | [1.769, 8.099] | 0.001 |
| Age 20–29 ***X*** 2002–2005 | 5.464 | [2.615, 11.420] | < 0.001 |
| Age 20–29 ***X*** 2005–2009 | 2.787 | [1.410, 5.506] | 0.003 |
| Age 30–39 ***X*** 1998–2001 | 2.659 | [1.264, 5.594] | 0.01 |
| Age 30–39 ***X*** 2002–2005 | 4.919 | [2.399, 10.089] | < 0.001 |
| Age 30–39 ***X*** 2005–2009 | 2.712 | [1.402, 5.247] | 0.003 |
| Age 40–49 ***X*** 1998–2001 | 3.207 | [1.492, 6.895] | 0.003 |
| Age 40–49 ***X*** 2002–2005 | 4.244 | [2.021, 8.911] | < 0.001 |
| Age 40–49 ***X*** 2005–2009 | 2.413 | [1.216, 4.787] | 0.012 |
| Age 50–59 ***X*** 1998–2001 | 2 | [0.920, 4.344] | 0.08 |
| Age 50–59 ***X*** 2002–2005 | 3.311 | [1.577, 6.951] | 0.002 |
| Age 50–59 ***X*** 2005–2009 | 2.12 | [1.072, 4.194] | 0.031 |
| Age 60–69 ***X*** 1998–2001 | 1.461 | [0.700, 3.050] | 0.312 |
| Age 60–69 ***X*** 2002–2005 | 1.443 | [0.703, 2.959] | 0.317 |
| Age 60–69 ***X*** 2005–2009 | 0.918 | [0.475, 1.775] | 0.799 |
| Age 70–79 ***X*** 1998–2001 | 1.113 | [0.537, 2.304] | 0.774 |
| Age 70–79 ***X*** 2002–2005 | 0.76 | [0.372, 1.552] | 0.451 |
| Age 70–79 ***X*** 2005–2009 | 0.6 | [0.313, 1.149] | 0.123 |
| Age 80+ ***X*** 1998–2001 | 0.982 | [0.466, 2.073] | 0.963 |
| Age 80+ ***X*** 2002–2005 | 0.811 | [0.392, 1.678] | 0.573 |
| Age 80+ ***X*** 2005–2009 | 0.52 | [0.269, 1.005] | 0.052 |
| *Interactions between Sex, Age, and Time* | |  |  |
| Male ***X*** age 10–19 ***X*** 1998–2001 | 0.687 | [0.210, 2.255] | 0.536 |
| Male ***X*** age 10–19 ***X*** 2002–2005 | 0.303 | [0.103, 0.896] | 0.031 |
| Male ***X*** age 10–19 ***X*** 2005–2009 | 0.522 | [0.183, 1.495] | 0.226 |
| Male ***X*** age 20–29 ***X*** 1998–2001 | 0.316 | [0.107, 0.931] | 0.037 |
| Male ***X*** age 20–29 ***X*** 2002–2005 | 0.189 | [0.070, 0.511] | 0.001 |
| Male ***X*** age 20–29 ***X*** 2005–2009 | 0.264 | [0.102, 0.686] | 0.006 |
| Male ***X*** age 30–39 ***X*** 1998–2001 | 0.547 | [0.190, 1.574] | 0.263 |
| Male ***X*** age 30–39 ***X*** 2002–2005 | 0.278 | [0.105, 0.736] | 0.01 |
| Male ***X*** age 30–39 ***X*** 2005–2009 | 0.475 | [0.188, 1.199] | 0.115 |
| Male ***X*** age 40–49 ***X*** 1998–2001 | 0.396 | [0.136, 1.156] | 0.09 |
| Male ***X*** age 40–49 ***X*** 2002–2005 | 0.249 | [0.093, 0.671] | 0.006 |
| Male ***X*** age 40–49 ***X*** 2005–2009 | 0.432 | [0.168, 1.110] | 0.081 |
| Male ***X*** age 50–59 ***X*** 1998–2001 | 0.425 | [0.143, 1.258] | 0.122 |
| Male ***X*** age 50–59 ***X*** 2002–2005 | 0.247 | [0.091, 0.665] | 0.006 |
| Male ***X*** age 50–59 ***X*** 2005–2009 | 0.322 | [0.125, 0.829] | 0.019 |
| Male ***X*** age 60–69 ***X*** 1998–2001 | 0.539 | [0.187, 1.553] | 0.252 |
| Male ***X*** age 60–69 ***X*** 2002–2005 | 0.517 | [0.195, 1.372] | 0.185 |
| Male ***X*** age 60–69 ***X*** 2005–2009 | 0.705 | [0.278, 1.788] | 0.462 |
| Male ***X*** age 70–79 ***X*** 1998–2001 | 0.902 | [0.317, 2.566] | 0.847 |
| Male ***X*** age 70–79 ***X*** 2002–2005 | 0.691 | [0.260, 1.835] | 0.459 |
| Male ***X*** age 70–79 ***X*** 2005–2009 | 0.734 | [0.290, 1.854] | 0.513 |
| Male ***X*** age 80+ ***X*** 1998–2001 | 0.878 | [0.297, 2.594] | 0.814 |
| Male ***X*** age 80+ ***X*** 2002–2005 | 0.621 | [0.227, 1.698] | 0.353 |
| Male ***X*** age 80+ ***X*** 2005–2009 | 0.924 | [0.356, 2.400] | 0.871 |

^a^ Logistic regression of adult death on sex, age, and time period. Unit of analysis is “person-year.” Explanatory variables are defined at beginning of each year.
