# Supplementary material for: The Unfolding Counter-Transition in Rural South Africa: Mortality and Cause of Death, 1994–2009
Source: PLoS One. 2014 Jun 24;9(6):e100420. doi: 10.1371/journal.pone.0100420 (PMC4068997; doi:10.1371/journal.pone.0100420)
Supplement: Table S3 — Logistic regression of all-cause mortality by household SES, Agincourt, South Africa, 2001–2009. (DOCX) [file pone.0100420.s003.docx]

| Variable | Odds Ratio | 95% CI | p-value |
| --- | --- | --- | --- |
| *Sex* |  |  |  |
| Male | 1.463 | [1.387, 1.544] | < 0.001 |
| *10-Year Age Groups* |  |  |  |
| 5–9 | 1.000 | – | – |
| 10–19 | 1.332 | [0.814, 2.179] | 0.254 |
| 20–29 | 6.601 | [4.245, 10.263] | < 0.001 |
| 30–39 | 15.372 | [9.967, 23.708] | < 0.001 |
| 40–49 | 18.955 | [12.246, 29.340] | < 0.001 |
| 50–59 | 22.272 | [14.295, 34.701] | < 0.001 |
| 60–69 | 30.579 | [19.558, 47.810] | < 0.001 |
| 70–79 | 36.081 | [23.004, 56.591] | < 0.001 |
| 80+ | 63.232 | [39.943, 100.098] | < 0.001 |
| *Time Period*­ |  |  |  |
| 2001–2002 | 1.000 | – | – |
| 2003–2004 | 1.415 | [1.298, 1.543] | < 0.001 |
| 2005–2006 | 1.625 | [1.491, 1.770] | < 0.001 |
| 2007–2008 | 1.604 | [1.471, 1.749] | < 0.001 |
| 2009 | 1.621 | [1.454, 1.807] | < 0.001 |
| *SES Quintiles* |  |  |  |
| 1^st^ (lowest) | 1.000 | – | – |
| 2^nd^ | 0.574 | [0.284, 1.161] | 0.122 |
| 3^rd^ | 0.911 | [0.493, 1.684] | 0.767 |
| 4^th^ | 0.938 | [0.507, 1.734] | 0.838 |
| 5^th ­^(highest) | 0.729 | [0.373, 1.427] | 0.357 |
| *Interactions Between SES and Age* |  |  |  |
| 2^nd^ quintile ***X*** age 10–19 | 1.019 | [0.449, 2.309] | 0.965 |
| 2^nd^ quintile ***X*** age 20–29 | 1.307 | [0.628, 2.724] | 0.474 |
| 2^nd^ quintile ***X*** age 30–39 | 1.601 | [0.778, 3.295] | 0.201 |
| 2^nd^ quintile ***X*** age 40–49 | 1.449 | [0.700, 3.000] | 0.317 |
| 2^nd^ quintile ***X*** age 50–59 | 1.485 | [0.712, 3.099] | 0.292 |
| 2^nd^ quintile ***X*** age 60–69 | 1.437 | [0.684, 3.017] | 0.338 |
| 2^nd^ quintile ***X*** age 70–79 | 1.392 | [0.661, 2.933] | 0.384 |
| 2^nd^ quintile ***X*** age 80+ | 1.895 | [0.894, 4.020] | 0.095 |
| 3^rd^ quintile ***X*** age 10–19 | 0.79 | [0.381, 1.637] | 0.526 |
| 3^rd^ quintile ***X*** age 20–29 | 0.723 | [0.378, 1.384] | 0.328 |
| 3^rd^ quintile ***X*** age 30–39 | 0.699 | [0.369, 1.324] | 0.272 |
| 3^rd^ quintile ***X*** age 40–49 | 0.653 | [0.343, 1.246] | 0.197 |
| 3^rd^ quintile ***X*** age 50–59 | 0.715 | [0.372, 1.377] | 0.316 |
| 3^rd^ quintile ***X*** age 60–69 | 0.692 | [0.357, 1.342] | 0.276 |
| 3^rd^ quintile ***X*** age 70–79 | 0.773 | [0.396, 1.508] | 0.45 |
| 3^rd^ quintile ***X*** age 80+ | 1.247 | [0.637, 2.438] | 0.52 |
| 4^th^ quintile ***X*** age 10–19 | 0.554 | [0.261, 1.174] | 0.123 |
| 4^th^ quintile ***X*** age 20–29 | 0.637 | [0.332, 1.220] | 0.174 |
| 4^th^ quintile ***X*** age 30–39 | 0.594 | [0.313, 1.127] | 0.111 |
| 4^th^ quintile ***X*** age 40–49 | 0.611 | [0.320, 1.165] | 0.135 |
| 4^th^ quintile ***X*** age 50–59 | 0.671 | [0.349, 1.290] | 0.232 |
| 4^th^ quintile ***X*** age 60–69 | 0.555 | [0.285, 1.081] | 0.083 |
| 4^th^ quintile ***X*** age 70–79 | 0.603 | [0.307, 1.184] | 0.142 |
| 4^th^ quintile ***X*** age 80+ | 0.982 | [0.499, 1.935] | 0.959 |
| 5^th^ quintile ***X*** age 10–19 | 0.744 | [0.335, 1.653] | 0.468 |
| 5^th^ quintile ***X*** age 20–29 | 0.689 | [0.340, 1.396] | 0.301 |
| 5^th^ quintile ***X*** age 30–39 | 0.682 | [0.340, 1.367] | 0.281 |
| 5^th^ quintile ***X*** age 40–49 | 0.57 | [0.282, 1.153] | 0.118 |
| 5^th^ quintile ***X*** age 50–59 | 0.666 | [0.327, 1.355] | 0.262 |
| 5^th^ quintile ***X*** age 60–69 | 0.706 | [0.345, 1.445] | 0.341 |
| 5^th^ quintile ***X*** age 70–79 | 0.894 | [0.433, 1.843] | 0.761 |
| 5^th^ quintile ***X*** age 80+ | 1.213 | [0.584, 2.520] | 0.604 |

^a^ Logistic regression of adult death on sex, age, time period, and household SES. Unit of analysis is “person-year.” Explanatory variables are defined at beginning of each year.
