# Supplementary material for: The Unfolding Counter-Transition in Rural South Africa: Mortality and Cause of Death, 1994–2009
Source: PLoS One. 2014 Jun 24;9(6):e100420. doi: 10.1371/journal.pone.0100420 (PMC4068997; doi:10.1371/journal.pone.0100420)
Supplement: Table S4 — Multinomial logistic regression of cause-specific mortality, Agincourt, South Africa, 1994–2009. (DOCX) [file pone.0100420.s004.docx]

| Variable | Odds Ratio | 95% CI | p-value |
| --- | --- | --- | --- |
| **HIV/TB** |  |  |  |
| *Sex* |  |  |  |
| Male | 0.983 | [0.549, 1.762] | 0.954 |
| *10-Year Age Groups* |  |  |  |
| 5–9 | 1.000 | – | – |
| 10–19 | 0.879 | [0.328, 2.357] | 0.798 |
| 20–29 | 7.12 | [3.194,15.874] | < 0.001 |
| 30–39 | 11.519 | [5.254, 25.254] | < 0.001 |
| 40–49 | 13.552 | [6.147, 29.875] | < 0.001 |
| 50–59 | 12.793 | [5.631, 29.061] | < 0.001 |
| 60–69 | 17.34 | [7.686, 39.119] | < 0.001 |
| 70–79 | 26.147 | [11.467, 59.619] | < 0.001 |
| 80+ | 54.626 | [22.474, 132.771] | < 0.001 |
| *Time Period* |  |  |  |
| 1994–1997 | 1.000 | – | – |
| 1998–2001 | 1.123 | [0.418, 3.020] | 0.818 |
| 2002–2005 | 2.336 | [0.982, 5.559] | 0.055 |
| 2006–2009 | 3.276 | [1.429, 7.510] | 0.005 |
| *Interactions between Sex and Age* | |  |  |
| Male ***X*** age 10–19 | 0.775 | [0.390, 1.540] | 0.467 |
| Male ***X*** age 20–29 | 0.448 | [0.252, 0.796] | 0.006 |
| Male ***X*** age 30–39 | 0.979 | [0.557, 1.719] | 0.94 |
| Male ***X*** age 40–49 | 1.503 | [0.851, 2.652] | 0.16 |
| Male ***X*** age 50–59 | 1.455 | [0.813, 2.602] | 0.206 |
| Male ***X*** age 60–69 | 1.898 | [1.049, 3.436] | 0.034 |
| Male ***X*** age 70–79 | 2.232 | [1.215, 4.101] | 0.01 |
| Male ***X*** age 80+ | 1.904 | [1.020, 3.555] | 0.043 |
| *Interactions between Sex and Time* | |  |  |
| Male ***X*** 1998–2001 | 0.934 | [0.714, 1.223] | 0.62 |
| Male ***X*** 2002–2005 | 0.926 | [0.724, 1.185] | 0.543 |
| Male ***X*** 2006–2009 | 1.069 | [0.834, 1.371] | 0.597 |
| *Interactions between Age and Time* | |  |  |
| Age 10–19 ***X*** 1998–2001 | 1.024 | [0.282, 3.723] | 0.971 |
| Age 10–19 ***X*** 2002–2005 | 1.68 | [0.558, 5.052] | 0.356 |
| Age 10–19 ***X*** 2006–2009 | 0.818 | [0.274, 2.440] | 0.719 |
| Age 20–29 ***X*** 1998–2001 | 2.328 | [0.824, 6.573] | 0.111 |
| Age 20–29 ***X*** 2002–2005 | 2.21 | [0.885, 5.518] | 0.089 |
| Age 20–29 ***X*** 2006–2009 | 1.047 | [0.433, 2.529] | 0.919 |
| Age 30–39 ***X*** 1998–2001 | 2.211 | [0.797, 6.131] | 0.127 |
| Age 30–39 ***X*** 2002–2005 | 2.374 | [0.968, 5.818] | 0.059 |
| Age 30–39 ***X*** 2006–2009 | 1.467 | [0.620, 3.467] | 0.383 |
| Age 40–49 ***X*** 1998–2001 | 1.774 | [0.636, 4.950] | 0.273 |
| Age 40–49 ***X*** 2002–2005 | 1.783 | [0.724, 4.392] | 0.208 |
| Age 40–49 ***X*** 2006–2009 | 1.051 | [0.442, 2.497] | 0.911 |
| Age 50–59 ***X*** 1998–2001 | 1.409 | [0.486, 4.087] | 0.528 |
| Age 50–59 ***X*** 2002–2005 | 1.845 | [0.728, 4.679] | 0.197 |
| Age 50–59 ***X*** 2006–2009 | 1.087 | [0.444, 2.664] | 0.855 |
| Age 60–69 ***X*** 1998–2001 | 1.324 | [0.460, 3.813] | 0.603 |
| Age 60–69 ***X*** 2002–2005 | 1.256 | [0.496, 3.182] | 0.63 |
| Age 60–69 ***X*** 2006–2009 | 0.662 | [0.269, 1.628] | 0.369 |
| Age 70–79 ***X*** 1998–2001 | 1.476 | [0.513, 4.247] | 0.471 |
| Age 70–79 ***X*** 2002–2005 | 0.587 | [0.227, 1.518] | 0.272 |
| Age 70–79 ***X*** 2006–2009 | 0.425 | [0.170, 1.058] | 0.066 |
| Age 80+ 19 ***X*** 1998–2001 | 1.382 | [0.451, 4.240] | 0.571 |
| Age 80+ 19 ***X*** 2002–2005 | 0.743 | [0.272, 2.030] | 0.562 |
| Age 80+ ***X*** 2006–2009 | 0.437 | [0.165, 1.155] | 0.095 |
| **Other Communicable Causes** | | |  |
| *Sex* |  |  |  |
| Male | 0.902 | [0.403, 2.023] | 0.803 |
| *10-Year Age Groups* |  |  |  |
| 5–9 | 1.000 | – | – |
| 10–19 | 1.495 | [0.545, 4.098] | 0.435 |
| 20–29 | 1.77 | [0.635, 4.937] | 0.275 |
| 30–39 | 1.639 | [0.561, 4.786] | 0.367 |
| 40–49 | 1.054 | [0.303, 3.673] | 0.934 |
| 50–59 | 3.355 | [1.107, 10.165] | 0.032 |
| 60–69 | 6.56 | [2.417, 17.801] | < 0.001 |
| 70–79 | 24.173 | [9.515, 61.412] | < 0.001 |
| 80+ | 73.242 | [27.735, 193.417] | < 0.001 |
| *Time Period* |  |  |  |
| 1994–1997 | 1.000 | – | – |
| 1998–2001 | 1.307 | [0.460, 3.710] | 0.615 |
| 2002–2005 | 0.166 | [0.020, 1.373] | 0.096 |
| 2006–2009 | 1.959 | [0.754, 5.092] | 0.168 |
| *Interactions between Sex and Age* | |  |  |
| Male ***X*** age 10–19 | 0.41 | [0.166, 1.014] | 0.054 |
| Male ***X*** age 20–29 | 0.578 | [0.236, 1.416] | 0.23 |
| Male ***X*** age 30–39 | 1.148 | [0.480, 2.747] | 0.756 |
| Male ***X*** age 40–49 | 2.031 | [0.816, 5.056] | 0.128 |
| Male ***X*** age 50–59 | 1.375 | [0.549, 3.440] | 0.497 |
| Male ***X*** age 60–69 | 2.319 | [0.954, 5.636] | 0.063 |
| Male ***X*** age 70–79 | 1.328 | [0.568, 3.103] | 0.513 |
| Male ***X*** age 80+ | 0.92 | [0.389, 2.176] | 0.85 |
| *Interactions between Sex and Time* | |  |  |
| Male ***X*** 1998–2001 | 0.894 | [0.530, 1.508] | 0.676 |
| Male ***X*** 2002–2005 | 1.067 | [0.623, 1.828] | 0.812 |
| Male ***X*** 2006–2009 | 1.608 | [0.992, 2.606] | 0.054 |
| *Interactions between Age and Time* | |  |  |
| Age 10–19 ***X*** 1998–2001 | 0.734 | [0.201, 2.684] | 0.64 |
| Age 10–19 ***X*** 2002–2005 | 6.674 | [0.717, 62.148] | 0.095 |
| Age 10–19 ***X*** 2006–2009 | 0.969 | [0.306, 3.064] | 0.957 |
| Age 20–29 ***X*** 1998–2001 | 0.882 | [0.241, 3.224] | 0.849 |
| Age 20–29 ***X*** 2002–2005 | 5.988 | [0.634, 56.537] | 0.118 |
| Age 20–29 ***X*** 2006–2009 | 0.808 | [0.252, 2.591] | 0.72 |
| Age 30–39 ***X*** 1998–2001 | 1.142 | [0.306, 4.258] | 0.843 |
| Age 30–39 ***X*** 2002–2005 | 12.359 | [1.323, 115.444] | 0.027 |
| Age 30–39 ***X*** 2006–2009 | 1.12 | [0.342, 3.671] | 0.852 |
| Age 40–49 ***X*** 1998–2001 | 2.084 | [0.493, 8.812] | 0.318 |
| Age 40–49 ***X*** 2002–2005 | 11.759 | [1.131, 122.309] | 0.039 |
| Age 40–49 ***X*** 2006–2009 | 1.797 | [0.477, 6.766] | 0.386 |
| Age 50–59 ***X*** 1998–2001 | 1.149 | [0.297, 4.448] | 0.84 |
| Age 50–59 ***X*** 2002–2005 | 6.74 | [0.686, 66.220] | 0.102 |
| Age 50–59 ***X*** 2006–2009 | 0.869 | [0.254, 2.968] | 0.822 |
| Age 60–69 ***X*** 1998–2001 | 0.729 | [0.209, 2.544] | 0.62 |
| Age 60–69 ***X*** 2002–2005 | 5.534 | [0.604, 50.702] | 0.13 |
| Age 60–69 ***X*** 2006–2009 | 0.586 | [0.189, 1.817] | 0.354 |
| Age 70–79 ***X*** 1998–2001 | 0.813 | [0.257, 2.578] | 0.726 |
| Age 70–79 ***X*** 2002–2005 | 2.947 | [0.329, 26.435] | 0.334 |
| Age 70–79 ***X*** 2006–2009 | 0.347 | [0.117, 1.025] | 0.055 |
| Age 80+ ***X*** 1998–2001 | 0.774 | [0.233, 2.567] | 0.675 |
| Age 80+ ***X*** 2002–2005 | 4.507 | [0.503, 40.372] | 0.178 |
| Age 80+ ***X*** 2006–2009 | 0.294 | [0.096, 0.902] | 0.032 |
| **Noncommunicable Causes** | | |  |
| *Sex* |  |  |  |
| Male | 1.864 | [0.539, 6.440] | 0.325 |
| *10-Year Age Groups* |  |  |  |
| 5–9 | 1.000 | – | – |
| 10–19 | 1.332 | [0.284, 6.256] | 0.716 |
| 20–29 | 3.046 | [0.725, 12.805] | 0.128 |
| 30–39 | 8.295 | [2.148, 32.029] | 0.002 |
| 40–49 | 7.504 | [1.878, 29.978] | 0.004 |
| 50–59 | 34.396 | [9.255, 127.835] | < 0.001 |
| 60–69 | 79.44 | [21.780, 289.749] | < 0.001 |
| 70–79 | 208.437 | [57.514, 755.401] | < 0.001 |
| 80+ | 528.304 | [143.858, 1940.144] | < 0.001 |
| *Time Period* |  |  |  |
| 1994–1997 | 1.000 | – | – |
| 1998–2001 | 0.603 | [0.109, 3.332] | 0.562 |
| 2002–2005 | 0.288 | [0.032, 2.595] | 0.267 |
| 2006–2009 | 1.442 | [0.356, 5.832] | 0.608 |
| *Interactions between Sex and Age* | |  |  |
| Male ***X*** age 10–19 | 0.544 | [0.129, 2.287] | 0.406 |
| Male ***X*** age 20–29 | 0.495 | [0.137, 1.781] | 0.282 |
| Male ***X*** age 30–39 | 0.743 | [0.210, 2.632] | 0.645 |
| Male ***X*** age 40–49 | 1.046 | [0.295, 3.709] | 0.945 |
| Male ***X*** age 50–59 | 1.133 | [0.323, 3.971] | 0.845 |
| Male ***X*** age 60–69 | 1.255 | [0.360, 4.374] | 0.721 |
| Male ***X*** age 70–79 | 1.078 | [0.311, 3.743] | 0.906 |
| Male ***X*** age 80+ | 0.765 | [0.219, 2.670] | 0.674 |
| *Interactions between Sex and Time* | |  |  |
| Male ***X*** 1998–2001 | 0.85 | [0.627, 1.151] | 0.294 |
| Male ***X*** 2002–2005 | 1.075 | [0.811, 1.426] | 0.614 |
| Male ***X*** 2006–2009 | 0.918 | [0.699, 1.204] | 0.535 |
| *Interactions between Age and Time* | |  |  |
| Age 10–19 ***X*** 1998–2001 | 1.95 | [0.259, 14.685] | 0.517 |
| Age 10–19 ***X*** 2002–2005 | 3.53 | [0.305, 40.825] | 0.313 |
| Age 10–19 ***X*** 2006–2009 | 0.953 | [0.166, 5.455] | 0.957 |
| Age 20–29 ***X*** 1998–2001 | 3.877 | [0.598, 25.151] | 0.156 |
| Age 20–29 ***X*** 2002–2005 | 14.871 | [1.483, 149.093] | 0.022 |
| Age 20–29 ***X*** 2006–2009 | 3.162 | [0.665, 15.029] | 0.148 |
| Age 30–39 ***X*** 1998–2001 | 2.219 | [0.369, 13.349] | 0.384 |
| Age 30–39 ***X*** 2002–2005 | 8.65 | [0.912, 82.013] | 0.06 |
| Age 30–39 ***X*** 2006–2009 | 2.475 | [0.569, 10.776] | 0.227 |
| Age 40–49 ***X*** 1998–2001 | 4.76 | [0.784, 28.917] | 0.09 |
| Age 40–49 ***X*** 2002–2005 | 9.461 | [0.976, 91.695] | 0.052 |
| Age 40–49 ***X*** 2006–2009 | 3.673 | [0.822, 16.420] | 0.089 |
| Age 50–59 ***X*** 1998–2001 | 2.104 | [0.367, 12.069] | 0.404 |
| Age 50–59 ***X*** 2002–2005 | 6.168 | [0.669, 56.886] | 0.108 |
| Age 50–59 ***X*** 2006–2009 | 1.421 | [0.339, 5.952] | 0.631 |
| Age 60–69 ***X*** 1998–2001 | 1.715 | [0.304, 9.667] | 0.541 |
| Age 60–69 ***X*** 2002–2005 | 4.323 | [0.473, 39.504] | 0.195 |
| Age 60–69 ***X*** 2006–2009 | 1.103 | [0.267, 4.554] | 0.893 |
| Age 70–79 ***X*** 1998–2001 | 1.395 | [0.249, 7.811] | 0.705 |
| Age 70–79 ***X*** 2002–2005 | 3.226 | [0.355, 29.359] | 0.299 |
| Age 70–79 ***X*** 2006–2009 | 0.859 | [0.210, 3.523] | 0.833 |
| Age 80+ 19 ***X*** 1998–2001 | 1.411 | [0.248, 8.017] | 0.698 |
| Age 80+ 19 ***X*** 2002–2005 | 3.011 | [0.328, 27.681] | 0.33 |
| Age 80+ ***X*** 2006–2009 | 0.785 | [0.189, 3.262] | 0.739 |
| **Injuries** |  |  |  |
| *Sex* |  |  |  |
| Male | 2.414 | [0.842, 6.922] | 0.101 |
| *10-Year Age Groups* |  |  |  |
| 5–9 | 1.000 | – | – |
| 10–19 | 1.593 | [0.399, 6.356] | 0.509 |
| 20–29 | 1.795 | [0.471, 6.840] | 0.392 |
| 30–39 | 1.875 | [0.472, 7.445] | 0.371 |
| 40–49 | 4.467 | [1.155, 17.282] | 0.03 |
| 50–59 | 8.252 | [2.060, 33.059] | 0.003 |
| 60–69 | 7.365 | [1.705, 31.822] | 0.007 |
| 70–79 | 15.923 | [3.933, 64.461] | < 0.001 |
| 80+ | 18.132 | [2.701, 121.746] | 0.003 |
| *Time Period* |  |  |  |
| 1994–1997 | 1.000 | – | – |
| 1998–2001 | 1.158 | [0.243, 5.521] | 0.854 |
| 2002–2005 | 3.896 | [1.112, 13.642] | 0.033 |
| 2006–2009 | 0.889 | [0.154, 5.127] | 0.895 |
| *Interactions between Sex and Age* | |  |  |
| Male ***X*** age 10–19 | 1.371 | [0.450, 4.181] | 0.579 |
| Male ***X*** age 20–29 | 4.155 | [1.414, 12.211] | 0.01 |
| Male ***X*** age 30–39 | 6.459 | [2.088, 19.985] | 0.001 |
| Male ***X*** age 40–49 | 2.823 | [0.956, 8.332] | 0.06 |
| Male ***X*** age 50–59 | 1.622 | [0.524, 5.019] | 0.401 |
| Male ***X*** age 60–69 | 1.381 | [0.435, 4.386] | 0.584 |
| Male ***X*** age 70–79 | 2.268 | [0.726, 7.085] | 0.159 |
| Male ***X*** age 80+ | 0.907 | [0.247, 3.340] | 0.884 |
| *Interactions between Sex and Time* | |  |  |
| Male ***X*** 1998–2001 | 0.584 | [0.303, 1.124] | 0.107 |
| Male ***X*** 2002–2005 | 0.571 | [0.311, 1.050] | 0.071 |
| Male ***X*** 2006–2009 | 0.671 | [0.355, 1.270] | 0.22 |
| *Interactions between Age and Time* | |  |  |
| Age 10–19 ***X*** 1998–2001 | 1.094 | [0.201, 5.968] | 0.917 |
| Age 10–19 ***X*** 2002–2005 | 0.508 | [0.128, 2.020] | 0.336 |
| Age 10–19 ***X*** 2006–2009 | 1.377 | [0.212, 8.970] | 0.738 |
| Age 20–29 ***X*** 1998–2001 | 1.397 | [0.281, 6.942] | 0.683 |
| Age 20–29 ***X*** 2002–2005 | 0.672 | [0.186, 2.432] | 0.545 |
| Age 20–29 ***X*** 2006–2009 | 1.921 | [0.324, 11.384] | 0.472 |
| Age 30–39 ***X*** 1998–2001 | 1.322 | [0.265, 6.604] | 0.734 |
| Age 30–39 ***X*** 2002–2005 | 0.522 | [0.142, 1.915] | 0.327 |
| Age 30–39 ***X*** 2006–2009 | 2.034 | [0.342, 12.078] | 0.435 |
| Age 40–49 ***X*** 1998–2001 | 1.64 | [0.323, 8.338] | 0.551 |
| Age 40–49 ***X*** 2002–2005 | 0.445 | [0.117, 1.700] | 0.237 |
| Age 40–49 ***X*** 2006–2009 | 2.194 | [0.362, 13.291] | 0.393 |
| Age 50–59 ***X*** 1998–2001 | 1.01 | [0.184, 5.557] | 0.991 |
| Age 50–59 ***X*** 2002–2005 | 0.32 | [0.078, 1.316] | 0.114 |
| Age 50–59 ***X*** 2006–2009 | 1.306 | [0.203, 8.403] | 0.779 |
| Age 60–69 ***X*** 1998–2001 | 1.13 | [0.181, 7.065] | 0.896 |
| Age 60–69 ***X*** 2002–2005 | 0.63 | [0.140, 2.825] | 0.546 |
| Age 60–69 ***X*** 2006–2009 | 2.245 | [0.320, 15.736] | 0.416 |
| Age 70–79 ***X*** 1998–2001 | 1.092 | [0.200, 5.969] | 0.919 |
| Age 70–79 ***X*** 2002–2005 | 0.374 | [0.091, 1.535] | 0.172 |
| Age 70–79 ***X*** 2006–2009 | 0.756 | [0.109, 5.242] | 0.777 |
| Age 80+ 19 ***X*** 1998–2001 | 0.744 | [0.063, 8.828] | 0.815 |
| Age 80+ 19 ***X*** 2002–2005 | 0.652 | [0.090, 4.706] | 0.671 |
| Age 80+ ***X*** 2006–2009 | 3.184 | [0.323, 31.419] | 0.321 |

^a^ Multinomial logistic regression of adult death by cause on sex, age, and time period. Unit of analysis is “person-year.” Explanatory variables are defined at beginning of each year. Referent group is surviving adults.
