# Supplementary material for: The Unfolding Counter-Transition in Rural South Africa: Mortality and Cause of Death, 1994–2009
Source: PLoS One. 2014 Jun 24;9(6):e100420. doi: 10.1371/journal.pone.0100420 (PMC4068997; doi:10.1371/journal.pone.0100420)
Supplement: Table S5 — Multinomial logistic regression of cause-specific mortality by household SES, Agincourt, South Africa, 2001–2009. (DOCX) [file pone.0100420.s005.docx]

| Variable | Odds Ratio | 95% CI | p-value |
| --- | --- | --- | --- |
| **HIV/TB** |  |  |  |
| *Sex* |  |  |  |
| Male | 1.076 | [0.998, 1.161] | 0.056 |
| *10-Year Age Groups* |  |  |  |
| 5–9 | 1.000 | – | – |
| 10–19 | 0.943 | [0.629, 1.413] | 0.776 |
| 20–29 | 8.366 | [5.934, 11.795] | < 0.001 |
| 30–39 | 21.847 | [15.570, 30.654] | < 0.001 |
| 40–49 | 23.453 | [16.666, 33.004] | < 0.001 |
| 50–59 | 22.437 | [15.842, 31.778] | < 0.001 |
| 60–69 | 22.459 | [15.710, 32.108] | < 0.001 |
| 70–79 | 18.931 | [13.038, 27.487] | < 0.001 |
| 80+ | 44.261 | [30.450, 64.336] | < 0.001 |
| *Time Period* |  |  |  |
| 2001–2002 | 1.000 | – | – |
| 2003–2004 | 1.597 | [1.418, 1.799] | < 0.001 |
| 2005–2006 | 1.685 | [1.494, 1.901] | < 0.001 |
| 2007–2008 | 1.545 | [1.365, 1.749] | < 0.001 |
| 2009 | 1.355 | [1.150, 1.596] | < 0.001 |
| *SES Quintiles* | |  |  |
| 1^st^ (lowest) | 1.000 | – | – |
| 2^nd^ | 0.846 | [0.761, 0.941] | 0.002 |
| 3^rd^ | 0.689 | [0.616, 0.771] | < 0.001 |
| 4^th^ | 0.536 | [0.475, 0.605] | < 0.001 |
| 5^th^ (highest) | 0.459 | [0.404, 0.521] | < 0.001 |
| **Other Communicable Causes** | | |  |
| *Sex* |  |  |  |
| Male | 1.414 | [1.146, 1.746] | 0.001 |
| *10-Year Age Groups* |  |  |  |
| 5–9 | 1.000 | – | – |
| 10–19 | 1.079 | [0.612, 1.904] | 0.792 |
| 20–29 | 1.304 | [0.736, 2.312] | 0.363 |
| 30–39 | 2.9 | [1.679, 5.010] | < 0.001 |
| 40–49 | 3.483 | [1.982, 6.121] | < 0.001 |
| 50–59 | 4.675 | [2.625, 8.327] | < 0.001 |
| 60–69 | 8.259 | [4.669, 14.609] | < 0.001 |
| 70–79 | 11.877 | [6.706, 21.036] | < 0.001 |
| 80+ | 37.699 | [21.806, 65.174] | < 0.001 |
| *Time Period* |  |  |  |
| 2001–2002 | 1.000 | – | – |
| 2003–2004 | 1.24 | [0.858, 1.794] | 0.253 |
| 2005–2006 | 1.731 | [1.217, 2.462] | 0.002 |
| 2007–2008 | 2.122 | [1.509, 2.984] | < 0.001 |
| 2009 | 2.154 | [1.430, 3.246] | < 0.001 |
| *SES Quintiles* | |  |  |
| 1^st^ (lowest) | 1.000 | – | – |
| 2^nd^ | 0.741 | [0.537, 1.021] | 0.067 |
| 3^rd^ | 0.648 | [0.464, 0.903] | 0.01 |
| 4^th^ | 0.712 | [0.515, 0.984] | 0.039 |
| 5^th^ (highest) | 0.687 | [0.496, 0.952] | 0.024 |
| **Noncommunicable Causes** | | |  |
| *Sex* |  |  |  |
| Male | 1.733 | [1.550, 1.938] | < 0.001 |
| *10-Year Age Groups* |  |  |  |
| 5–9 | 1.000 | – | – |
| 10–19 | 1.379 | [0.501, 3.793] | 0.534 |
| 20–29 | 11.574 | [4.712, 28.429] | < 0.001 |
| 30–39 | 25.8 | [10.569, 62.978] | < 0.001 |
| 40–49 | 38.95 | [15.952, 95.103] | < 0.001 |
| 50–59 | 91.112 | [37.510, 221.310] | < 0.001 |
| 60–69 | 165.758 | [68.305, 402.251] | < 0.001 |
| 70–79 | 276.408 | [114.021, 670.068] | < 0.001 |
| 80+ | 554.367 | [228.376, 1345.686] | < 0.001 |
| *Time Period* |  |  |  |
| 2001–2002 | 1.000 | – | – |
| 2003–2004 | 1.327 | [1.095, 1.610] | 0.004 |
| 2005–2006 | 1.843 | [1.532, 2.217] | < 0.001 |
| 2007–2008 | 1.977 | [1.645, 2.375] | < 0.001 |
| 2009 | 2.583 | [2.090, 3.194] | < 0.001 |
| *SES Quintiles* | |  |  |
| 1^st^ (lowest) | 1.000 | – | – |
| 2^nd^ | 0.825 | [0.704, 0.968] | 0.018 |
| 3^rd^ | 0.655 | [0.553, 0.776] | < 0.001 |
| 4^th^ | 0.559 | [0.468, 0.666] | < 0.001 |
| 5^th^ (highest) | 0.502 | [0.419, 0.601] | < 0.001 |
| **Injuries** |  |  |  |
| *Sex* |  |  |  |
| Male | 3.74 | [2.929, 4.776] | < 0.001 |
| *10-Year Age Groups* |  |  |  |
| 5–9 | 1.000 | – | – |
| 10–19 | 1.209 | [0.622, 2.347] | 0.576 |
| 20–29 | 4.415 | [2.417, 8.063] | < 0.001 |
| 30–39 | 5.881 | [3.195, 10.827] | < 0.001 |
| 40–49 | 6.838 | [3.660, 12.773] | < 0.001 |
| 50–59 | 5.464 | [2.777, 10.750] | < 0.001 |
| 60–69 | 8.251 | [4.143, 16.434] | < 0.001 |
| 70–79 | 11.652 | [5.757, 23.583] | < 0.001 |
| 80+ | 21.639 | [10.407, 44.992] | < 0.001 |
| *Time Period* |  |  |  |
| 2001–2002 | 1.000 | – | – |
| 2003–2004 | 1.135 | [0.825, 1.563] | 0.436 |
| 2005–2006 | 1.309 | [0.956, 1.793] | 0.093 |
| 2007–2008 | 1.061 | [0.764, 1.475] | 0.723 |
| 2009 | 1.009 | [0.657, 1.549] | 0.969 |
| *SES Quintiles* | |  |  |
| 1^st^ (lowest) | 1.000 | – | – |
| 2^nd^ | 1.039 | [0.753, 1.432] | 0.816 |
| 3^rd^ | 0.812 | [0.576, 1.144] | 0.234 |
| 4^th^ | 0.976 | [0.702, 1.355] | 0.883 |
| 5^th^ (highest) | 0.861 | [0.612, 1.211] | 0.39 |

^a^ Multinomial logistic regression of adult death by cause on sex, age, and time period. Unit of analysis is “person-year.” Explanatory variables are defined at beginning of each year. Referent group is surviving adults.
